# Effects of *Lactobacillus acidophilus* on hepatic and microbial dysregulation in MASLD and diabetes

**DOI:** 10.1080/19490976.2026.2734705

**Published:** 2026-09-19

**Authors:** Hee Jin Park, In Gyu Park, Jeong Ha Park, Hyun Joon Park, Jin Ju Jung, Sang Hak Han, Ki Tae Suk

**Affiliations:** a Institute for Liver and Digestive Diseases, Hallym University, Chuncheon, Republic of Korea; b Department of Pathology, Hallym University College of Medicine, Chuncheon, Republic of Korea

**Keywords:** *Lactobacillus acidophilus*, metabolic-associated steatotic liver disease, diabetes, gut microbiota, probiotics

## Abstract

Gut microbial dysbiosis has been implicated in metabolic dysfunction-associated steatotic liver disease (MASLD) and diabetes, but the metabolic effects of candidate probiotic strains across this disease continuum remain incompletely understood. Based on the validation of disease-associated microbial patterns in a clinical cohort, *Lactobacillus acidophilus* as selected as a candidate strain, and its effects were systematically investigated across multiple complementary models, including Western diet, fructose–palmitate–cholesterol diet, high-fat diet, and leptin-deficient ob/ob mice. *L. acidophilus* supplementation attenuated hepatic steatosis and inflammatory injury in multiple complementary models and improved glucose tolerance in ob/ob mice. These effects were accompanied by reduced hepatic expression of pro-inflammatory cytokines, changes in AMPK-associated signaling, decreased expression of lipogenic (*Srebf1* and *Acc*) and gluconeogenic (*Pepck* and *G6pc*) genes, and increased *Ppara* expression, suggesting coordinated regulation of glucose and lipid metabolism. Transcriptomic profiling further indicated that *L. acidophilus* influenced broader metabolic networks, including altered expression of *Lpin1* and enrichment of pathways related to nutrient sensing and metabolic regulation. In addition, *L. acidophilus* supplementation altered gut microbial community structure in mouse models, while human cohort analyzes revealed context-dependent microbial patterns involving *Lactobacillus*, *Akkermansia*, and butyrate-producing Firmicutes across metabolic disease states. Collectively, these findings suggest that *L. acidophilus* may improve metabolic dysfunction through coordinated hepatic metabolic regulation and gut microbial modulation, supporting its potential as a probiotic candidate for MASLD and diabetes-associated metabolic disease.

## Graphical abstract

**Figure:**
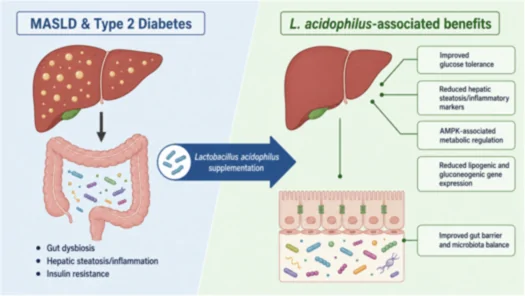

## Introduction

Metabolic dysfunction-associated steatotic liver disease (MASLD) is one of the most prevalent chronic liver diseases worldwide. Due to Western-style changes in eating habits and lack of exercise, its prevalence is also increasing along with obesity and metabolic syndrome in South Korea.^1^ MASLD and its progressive form, metabolic dysfunction-associated steatohepatitis (MASH), represent a spectrum of nonalcoholic fatty liver disease characterized by hepatic steatosis, inflammation, and varying degrees of fibrosis. The transition from MASLD to MASH is driven by metabolic stress, immune dysregulation, and intestinal-hepatic axis disturbance, and as it worsens, it can lead to cirrhosis and hepatocellular carcinoma.^2^ MASLD is closely associated with metabolic comorbidities such as obesity, hypertension, dyslipidemia, and particularly type 2 diabetes (T2D), with nearly 90% of affected individuals exhibiting features of metabolic syndrome.^3^

T2D, a key metabolic disorder, contributes significantly to MASLD progression via impaired hepatic glucose metabolism and systemic insulin resistance.^4^ ^,^ ^5^ Insulin resistance, a representative characteristic of T2D, was observed in most patients with MASLD.^6^ The prevalence of diabetes has been continuously increasing in recent years.^7^ Despite the increasing prevalence of T2D, current therapeutic-including lifestyle modification and pharmacological interventions-often show limited long-term adherence or efficacy in achieving sustained metabolic improvement. Certain antidiabetic drugs may improve MASLD by enhancing glucose uptake and insulin sensitivity; however, their effects on hepatic pathology remain indirect, and the underlying mechanisms are not fully understood.^8^ As an alternative, modulation of gut microbiome composition has emerged as a promising therapeutic strategy for metabolic diseases, including T2D and MASLD.^9^

Gut microbiota is known to have a major impact on human health and disease through interactions with the human body.^10^ In recent years, gut microbiota have emerged as important regulators of host metabolism, immunity, and liver function.^11^ ^,^ ^12^ Dysbiosis has been implicated in the pathogenesis of metabolic diseases including T2D and MASLD, primarily via its effects on chronic inflammation, insulin resistance, and intestinal barrier function.^13^ Consequently, microbiota-targeted interventions using pre-, pro-, post-, or parabiotics are being actively explored as novel therapeutic strategies.^14^ Probiotics are live microbial supplements that have beneficial effects on human health.^15^ Among them, *Lactobacillus* bacteria are most commonly used as a probiotic ingredient and inhabit the oral cavity, intestine, and vagina.^16^ Experiments using different strains belonging to this genus have shown beneficial effects in the prevention or treatment of various liver diseases.^17^ However, the strain-specific metabolic effects and antidiabetic mechanisms of *Lactobacillus* remain incompletely understood.

In our human cohort, *Lactobacillus* abundance showed a gradual decline with MASLD progression and diabetes. This pattern suggests a loss of potentially beneficial taxa during metabolic deterioration and provides a rationale for evaluating replenishment strategies. Given that *Lactobacillus* tends to decline in metabolic disorders and considering the known metabolic relevance of specific *Lactobacillus* strains, we focused on *L. acidophilus* as a candidate probiotic with potential benefits on lipid and glucose metabolism.

Here, we investigated the therapeutic potential of *L. acidophilus* in the context of T2D and diabetes-associated metabolic dysfunction. Given the strong bidirectional relationship between T2D and MASLD, we examined whether *L. acidophilus* could improve hepatic metabolic abnormalities across the MASLD–T2D continuum. We focused on its ability to modulate inflammation, lipid metabolism, and hepatic gluconeogenesis. Specifically, this study aimed to determine whether *L. acidophilus* modulates diabetes-associated metabolic dysfunction through regulation of hepatic AMPK signaling and gut microbial composition.

## Results

### Human microbiome profiling prioritizes L. acidophilus as a candidate species

**Figure 1.**
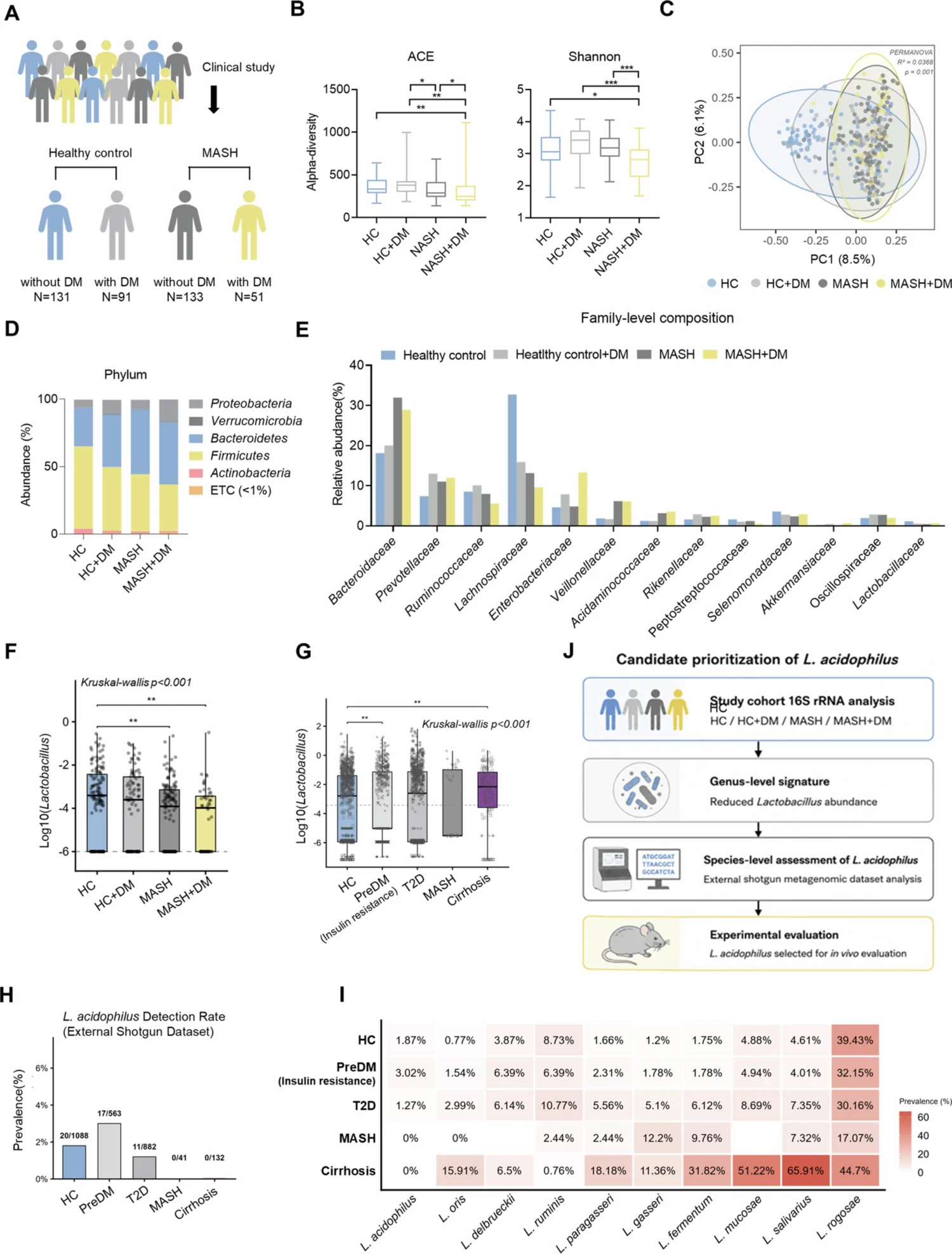
Human cohort gut microbiome profiling identifies liver disease–and DM associated taxa to guide candidate strain selection. (A) Schematic of the clinical study design and group allocation: healthy control without diabetes (HC, *n* = 131), healthy control with diabetes (HC + DM, *n* = 91), MASH without diabetes (MASH, *n* = 133), and MASH with diabetes (MASH + DM, *n* = 51). (B) The relative abundance of alpha diversity including the ACE index and Shannon. (C) Principal coordinate analysis (PCoA) based on Bray-Curtis dissimilarity of gut microbiome composition at the genus level (*n* = 406). Each point represents an individual sample, colored by group. Ellipses indicate 95% confidence intervals. Overall community difference was assessed by PERMANOVA (adonis2, 999 permutations): R^2^ = 0.037, *p* = 0.001. (D) Phylum-level community composition shown as relative abundance (%); taxa with <1% mean relative abundance was pooled as “ETC”. (E) Family-level mean relative abundance (%) of representative taxa across groups. (F) Genus-level *Lactobacillu*s relative abundance in the study cohort (*n* = 406). Log10-transformed relative abundance (pseudocount 10-6) is shown across four groups: HC (*n* = 131), HC + DM (*n* = 91), MASH (*n* = 133), and MASH + DM (*n* = 51). Boxes represent interquartile range with median line; individual data points are overlaid. Dashed line indicates the detection limit. Statistical comparison by Kruskal-Wallis test with pairwise Wilcoxon rank-sum tests (Benjamini-Hochberg corrected). **p < 0.01. (G) Genus-level *Lactobacillus* relative abundance in external shotgun metagenomics cohorts (9 studies, *n* = 2,706). Groups are ordered by metabolic disease progression: HC (*n* = 1,088), PreDM (*n* = 563), T2D (*n* = 882), NAFLD (*n* = 41), and Cirrhosis (*n* = 132). Statistical comparison by Wilcoxon rank-sum tests versus HC (Benjamini-Hochberg corrected). **p < 0.01. (H) Detection rate (prevalence) of L. acidophilus in external shotgun metagenomics cohorts. Numbers above bars indicate detected samples out of total samples per group. (I) Prevalence of the top 10 *Lactobacillus* species across disease groups in external shotgun metagenomics cohorts. Cell values represent the proportion of samples with detectable abundance (>0%) in each group. (J) Schematic overview of the *L. acidophilus* candidate prioritization process.

To identify disease- and diabetes-associated gut microbial signatures relevant to metabolic dysfunction, we first analyzed fecal microbiome profiles from a human cohort stratified by liver disease status and the presence of DM. DM, diabetes mellitus; in this study, refers to clinically diagnosed type 2 diabetes. As an initial step, we focused on DM-associated signatures in the advanced disease context by stratifying subjects into four groups: healthy control without DM (HC, *n* = 131), healthy control with DM (HC + DM, *n* = 91), MASH without DM (MASH, *n* = 133), and MASH with DM (MASH + DM, *n* = 51) (Figure 1A, Supplementary Table 1).

Baseline characteristics differed across the four study groups (Table 1). Compared with healthy controls, subjects with MASH, regardless of diabetes status, showed higher BMI and elevated liver injury markers, including AST, ALT, and GGT. HDL levels tended to be lower in diabetes-associated groups compared with the MASH group. Alpha diversity analysis revealed a progressive alteration of microbial diversity according to disease severity and diabetic status. Both ACE richness and Shannon diversity indices were significantly reduced in the MASH + DM groups compared with HC and HC + DM groups, indicating a pronounced loss of microbial diversity in patients with combined metabolic and hepatic dysfunction (Figure 1B). PCoA based on Bray-Curtis dissimilarity revealed a statistically significant difference in overall gut microbiome composition among the four groups (PERMANOVA R^2^ = 0.037, *p* = 0.001; Figure 1C). At the phylum level, all groups were dominated by Firmicutes and Bacteroidetes, with a relative increase in *Proteobacteria* observed in HC + DM and MASH + DM groups (Figure 1D). At the family level, *Bacteroidaceae* and *Lachnospiraceae* were the most abundant families across all groups. *Lactobacillaceae* showed visually lower abundance in MASH and MASH + DM groups compared to HC (Figure 1E), consistent with the genus-level findings described below (Figure 1F).

**Table 1.** Baseline characteristics of the four study groups (HC, HC + DM, MASH, MASH + DM).

Variables (mean [SEM])	Heathly control (*n = 131*)	Heathly control + DM (*n = 91*)	MASH (*n = 133*)	MASH + DM (*n = 51*)
Age (years)	48.5 (1.8)	68.2 (1.3)	46.7 (1.3)	57.1 (1.8)
BMI (kg/m^2^)	24.5 (0.3)	25.2 (0.4)	28.2 (0.4)	27.9 (0.5)
AST (IU/L)	26.5 (1.4)	33.1 (3.1)	51.5 (2.5)	50.6 (3.4)
ALT(IU/L)	30.1 (4.4)	33.1 (4.0)	70.0 (3.5)	64.8 (4.5)
Creatine (mg/dL)	0.8 (0)	0.9 (0)	0.8 (0)	0.9 (0)
Cholesterol (mg/dL)	186.6 (3.2)	142.7 (3.5)	186.9 (3.3)	163.6 (5.3)
γGTP (IU/L)	27.32 (2.5)	42.5 (5.9)	74.0 (6.9)	56.7 (4.3)
Triglyceride (mg/dL)	133.4 (9.6)	155.8 (7.5)	183.8 (14.2)	197.8 (24.9)
HDL (mg/dL)	19.3 (1.8)	43.3 (1.0)	46.9 (1.1)	43.1 (1.5)

In the expanded study cohort (*n* = 406), genus-level *Lactobacillus* relative abundance was significantly lower in MASH and MASH + DM groups compared to HC (Kruskal-Wallis *p* < 0.001; pairwise Wilcoxon, BH-corrected *p* < 0.01; Figure 1F). Internal shotgun metagenomic sequencing of a subset (*n* = 44) confirmed this genus-level depletion and identified *L. acidophilus* as the species showing the most consistent decrease across disease progression, with a significant negative correlation with serum GGT (Rho = −0.33, *p* = 0.02; Supplementary Figure 1B–D). To assess these findings in independent cohorts, we analyzed 2,706 shotgun metagenomics samples from 9 published studies (curatedMetagenomicData). To account for inter-study technical variation, study-level batch effects were adjusted by regressing out study as a covariate from log10-transformed relative abundances, preserving the original abundance scale. After batch correction, *Lactobacillus* abundance showed a decreasing trend in MASH compared to HC (*p* = 0.053), while cirrhosis exhibited significantly higher abundance (*p* < 0.01; Figure 1G). At the species level, *L. acidophilus* was not detected in any MASH (0/41) or cirrhosis (0/132) sample across external shotgun cohorts, while present at low prevalence in HC (1.87%), pre-diabetes (3.02%), and T2D (1.27%; Figure 1H). Among the 10 most prevalent *Lactobacillus* species, *L. acidophilus* was the only species absent in both MASH and cirrhosis groups. The remaining species, including *L. delbrueckii*, were maintained or elevated in disease groups (Figure 1I). Based on this species-specific pattern and the existing hepatoprotective literature,^18-22^ *L. acidophilus* was selected for in vivo evaluation (Figure 1J).

### L. acidophilus attenuates Western diet (WD)–induced hepatic steatosis and gut microbiota alterations

**Figure 2.**
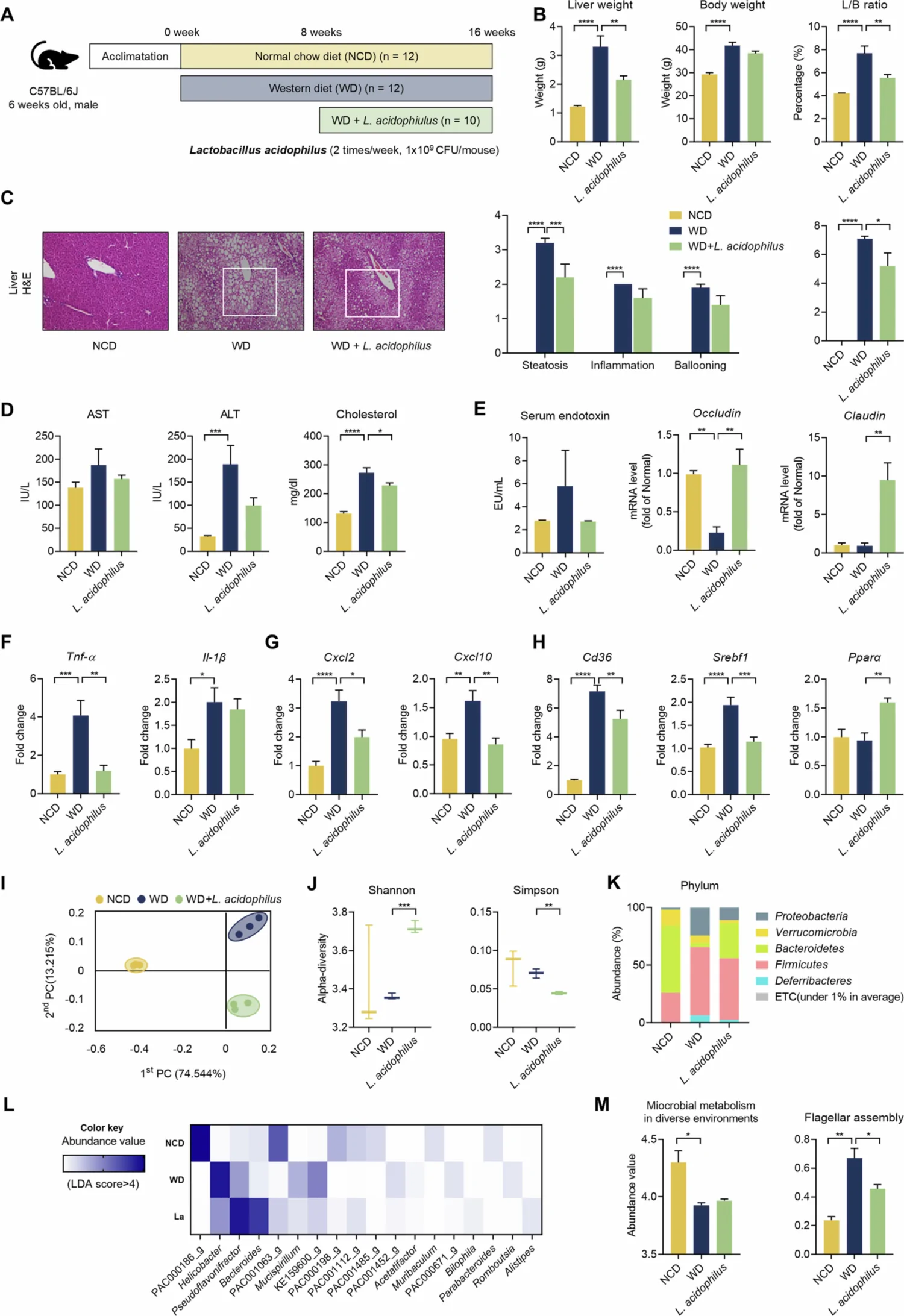
Effects of *L. acidophilus* on WD–induced hepatic injury and gut microbiota alterations. (A) The scheme shows the animal experiment in the WD model with the inclusion of the *L. acidophilus* treatment group. (B) Liver weight, body weight, and liver-to-body weight ratio following *L. acidophilus* treatment. (C) Representative H&E-stained liver sections. Steatosis, lobular inflammation, and hepatocellular ballooning were scored. (D) Serum biochemistry: AST, ALT and total cholesterol. (E) Serum endotoxin and intestinal tight junction gene expression. (F) Hepatic mRNA expression of pro-inflammatory genes and (G)chemokine genes. (H) Expression of lipid metabolism–related genes. (I) Principal coordinate analysis of gut microbiota. (J) Alpha-diversity (Shannon, Simpson). (K) Phylum composition. (L) Differential taxa (LDA > 4) presented as a heatmap. (M) Predicted microbial functional pathways (KEGG). Values are shown as mean ± SEM. Some error bars may not be visible when the SEM value is 0 or very small. Significant differences between groups are denoted as follows: *P < 0.05, **P < 0.01, ***P < 0.001. Abbreviations: ALT, alanine aminotransferase; AST, aspartate aminotransferase; *Tnf-α*, Tumor necrosis factor-alpha; *Il-1β*, Interleukin 1 beta; *Cxcl2*, Chemokine C-X-C motif ligand 2; *Cxcl10*, Chemokine C-X-C motif ligand 10; *Cd36*, Cluster of differentiation 36; *Srebf1*, Sterol regulatory element-binding transcription factor 1; *Pparα*, Peroxisome proliferator-activated receptor; LEfSe, Linear discriminant analysis effect Size.

We next evaluated whether *L. acidophilus* supplementation improves WD–induced hepatic steatosis in mice. Male C57BL/6J mice were fed a WD for 16 weeks to induce hepatic steatosis, and *L. acidophilus* was administered orally twice/week (1 × 10^9^ CFU/mouse) during the final 8 weeks (NCD *n* = 12, WD *n* = 12, and WD + *L. acidophilus* *n* = 10) (Figure 2A). WD feeding increased liver weight, body weight, and the liver-to-body weight ratio compared with normal chow diet (NCD), whereas *L. acidophilus* supplementation reduced liver weight and attenuated hepatic steatosis (Figure 2B and C). Histological inflammation and ballooning scores showed a decreasing pattern but did not reach statistical significance. Serum biochemistry was assessed to evaluate liver injury and AST and ALT showed nonsignificant downward trend (Figure 2D). Given that gut-derived endotoxin and impaired intestinal barrier function are associated with diet-induced liver injury,^23^ we measured serum endotoxin and intestinal tight junction markers. *L. acidophilus* supplementation tended to lower serum endotoxin levels and increased the expression of tight junction genes (*Occludin* and *Claudin*), consistent with improved barrier-associated markers (Figure 2E). To further characterize hepatic inflammatory responses, we quantified mRNA levels of pro-inflammatory cytokines (*Tnf-α*, *Il1β*) and chemokines (*Cxcl2*, *Cxcl10*). These inflammatory genes were markedly induced by WD feeding and were significantly reduced by *L. acidophilus* supplementation (Figure 2F and G).Regarding lipid metabolism, *L. acidophilus* significantly reduced the expression of *Cd36*, a mediator of long-chain fatty acid uptake, as well as *Srebf1*, a key regulator of lipogenesis.. In contrast, *L. acidophilus* restored *Ppara* expression, a key regulator of fatty acid *β*-oxidation, consistent with improved lipid catabolic programs in the liver (Figure 2H).

Collectively, these findings indicate that *L. acidophilus* modulates hepatic inflammatory responses and improves lipid metabolic markers under WD-induced metabolic stress. To further determine whether *L. acidophilus* supplementation modulates gut microbial composition in diet-induced hepatic steatosis, 16S rRNA sequencing was performed on fecal samples collected. Principal coordinate analysis (PCoA) based on *β*-diversity showed distinct clustering among groups, indicating that *L. acidophilus* supplementation altered the gut microbial community structure compared with WD feeding (Figure 2I). Consistently, *α*-diversity analyzes indicated that the WD-induced loss of microbial diversity was significantly restored by *L. acidophilus* treatment: the Shannon index increased (*p* < 0.0001), while the Simpson index decreased (*p* = 0.0021), reflecting recovery of microbial richness and evenness (Figure 2J). At the phylum level, WD feeding caused a characteristic dysbiosis, with a pronounced expansion of *Firmicutes* and loss of *Bacteroidetes. L. acidophilus* supplementation partially reversed these changes, restoring the F/B balance (NCD: 25/58%, WD: 59/3%, and *L. acidophilus*: 53/30%) (Figure 2K). To further characterize genus-level alterations induced by probiotic treatment, we examined the relative abundance of representative gut-associated taxa implicated in metabolic and immune homeostasis. The abundance of *Bacteroides*, one of the dominant genera in the healthy gut microbiota, was markedly reduced in WD-fed mice but was significantly restored following administration of *L. acidophilus*. Conversely, *Proteobacteria*, a phylum often associated with gut dysbiosis, was significantly elevated in WD–fed mice and effectively suppressed *L. acidophilus* supplementation. Other taxa, including *Akkermansia*, *Christensenellaceae*, and *Lachnospiraceae*, exhibited no significant changes in relative abundance following probiotic intervention (Supplementary Figure 2). To identify group-discriminatory taxa, the linear discriminant analysis effect size (LEfSe) was performed, revealing enrichment of dysbiosis-associated taxa in the WD group and partial restoration of beneficial taxa in the *L. acidophilus* treated group (Figure 2L). Functional inference of microbial communities predicted enhanced microbial metabolism in diverse environments and reduced flagellar assembly pathways following *L. acidophilus* supplementation (Figure 2M). Together, these data show that *L. acidophilus* supplementation attenuates WD-induced hepatic steatosis and inflammatory responses and is accompanied by improvements in gut microbial diversity and composition.

Evaluation of the protective effects of L. acidophilus on hepatic steatosis and inflammatory gene expression in high-fat diet (HFD) and fructose-palmitate-cholesterol (FPC) diet-induced modelsTo further examine the potential hepatoprotective effects of *L. acidophilus* observed in the WD model, we extended our investigation to two additional diet-induced models representing different stages of MASLD progression. The FPC diet model, which is known to elicit advanced steatohepatitis with fibrotic remodeling,^24^ was applied to explore the effects of *L. acidophilus* under more severe metabolic stress. In addition, a HFD model, reflecting early metabolic dysfunction and steatosis, was utilized to evaluate its possible preventive effects.^25^ These complementary settings enabled assessment of model- and stage-dependent hepatic and metabolic outcomes associated with *L. acidophilus* supplementation.

**Figure 3.**
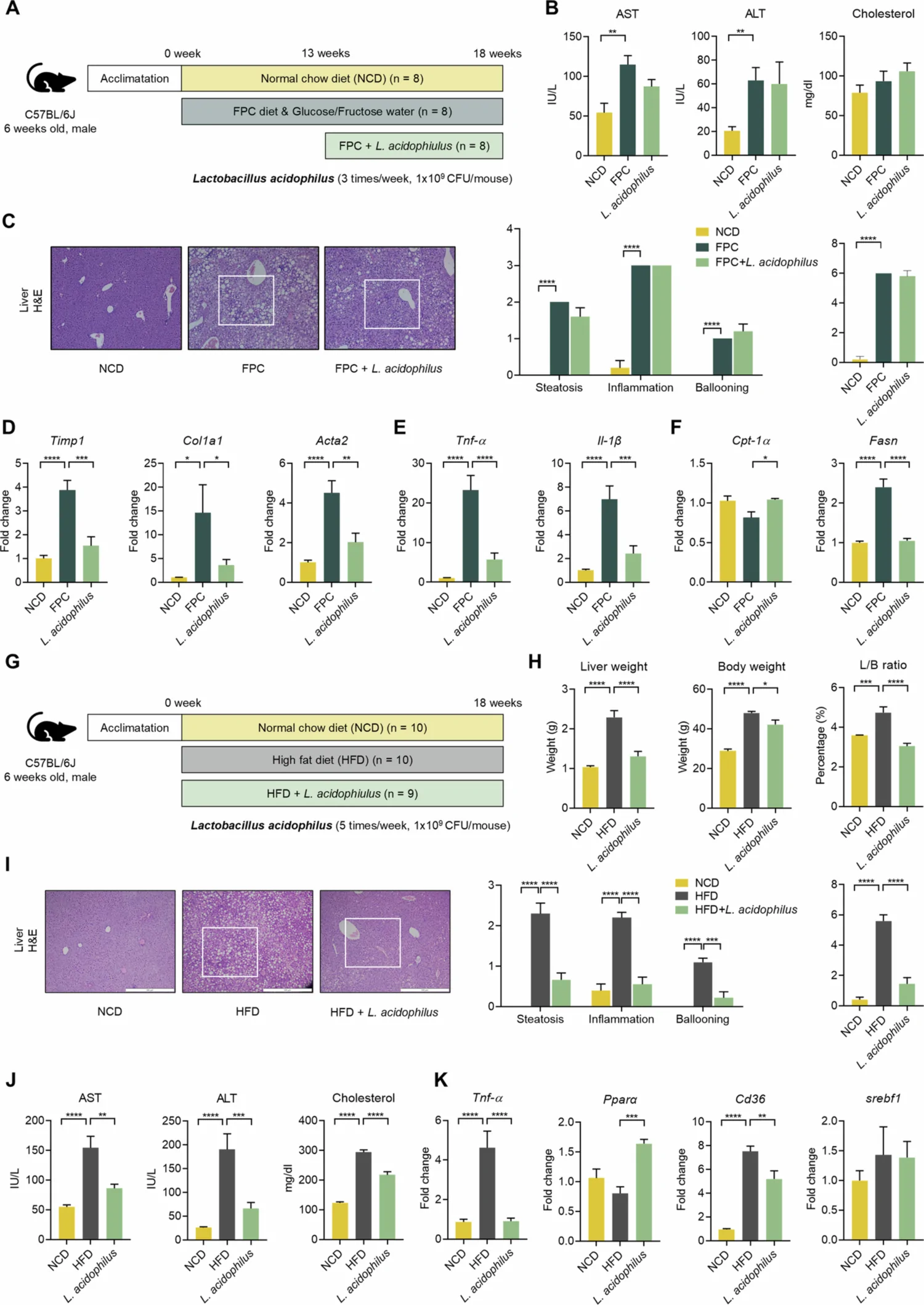
Effects of *L. acidophilus* on liver injury, inflammation, and metabolic dysfunction in FPC and HFD mouse models. (A) Experimental scheme for the FPC diet model. (B) Serum biochemistry in the FPC model: AST, ALT and total cholesterol. (C) Representative H&E-stained liver sections in the FPC model. Steatosis, lobular inflammation, and hepatocellular ballooning were scored. (D) Hepatic mRNA expression of fibrosis-related genes, (E) inflammatory cytokines and (F) lipid metabolism–related genes in the FPC model. (G) Experimental scheme for the HFD model. (H) Liver weight, body weight, and liver-to-body weight ratio in the HFD model. (I) Representative H&E-stained liver sections in the HFD model. Steatosis, lobular inflammation, and hepatocellular ballooning were scored. (J) Serum biochemistry in the HFD model: AST, ALT, and total cholesterol. (K) Hepatic mRNA expression of inflammation- and lipid metabolism–related genes in HFD model. Values are shown as mean ± SEM. Some error bars may not be visible when the SEM value is 0 or very small. Significant differences between groups are denoted as follows: *P < 0.05, **P < 0.01, ***P < 0.001. Abbreviations: *Timp1,* Tissue inhibitor of metalloproteinases 1; *Col1a1*, Collagen type I alpha 1 chain; *Acta2,* Actin alpha 2, smooth muscle; *Cpt-1α*, Carnitine palmitoyl transferase 1*α; Fasn*, Fatty acid synthase; *Tnf-α*, Tumor necrosis factor-alpha; *Pparα*, Peroxisome proliferator-activated receptor; *Cd36*, Cluster of differentiation 36; *Srebf1*, Sterol regulatory element-binding transcription factor 1.

In the FPC model (Figure 3A), mice were fed an FPC diet for 13 weeks, followed by 5 weeks of *L. acidophilus* supplementation, with 8 mice included in each group. Serum AST and ALT activities showed a nonsignificant downward trend, while total cholesterol levels and histological scores remained largely unchanged (Figure 3B and C). Hepatic mRNA expression of fibrosis-related genes, including tissue inhibitor of metalloproteinase 1, collagen type I alpha 1 chain and alpha smooth muscle actin (*Timp1, Col1a1,* and *Acta2*) was significantly upregulated in the FPC-fed group, whereas *L. acidophilus* treatment reduced their expression (Figure 3D). Similarly, pro-inflammatory cytokine genes (*Tnf-α* and *Il-1β*) were elevated in the FPC group but were significantly reduced by *L. acidophilus* (Figure 3E). Furthermore, *L. acidophilus* increased the expression of the fatty acid oxidation gene *Cpt-1α* while reducing the lipogenic gene *Fasn* (Figure 3F). Collectively, these findings suggest that *L. acidophilus* primarily modulated molecular markers associated with fibrosis, inflammation, and lipid metabolism in the FPC model, although these changes were not accompanied by overt histological or biochemical improvement.

In the HFD model (Figure 3G), mice received *L. acidophilus* supplementation throughout 18 weeks of HFD feeding (NCD *n* = 10, HFD *n* = 10, and HFD+ *L. acidophilus* *n* = 9). Compared with HFD-fed controls, *L. acidophilus* administration significantly reduced both liver and body weights, as well as the liver-to-body weight ratio (Figure 3H). Histological assessment showed that *L. acidophilus* attenuated hepatic steatosis, inflammatory infiltration, and ballooning degeneration (Figure 3I). Consistent with these findings, serum AST and ALT activities and hepatic triglyceride content were lower in *L. acidophilus*-treated mice (Figure 3J). Hepatic gene expression analysis revealed that *L. acidophilus* modulated key regulators of lipid metabolism and inflammation in HFD-fed mice. *L. acidophilus* reduced *Tnf-α* expression and increased *Ppara*, while downregulating the lipid uptake–associated gene *Cd36*; *Srebf1* expression was unchanged (Figure 3K). Collectively, these data suggest that *L. acidophilus* attenuates HFD-induced hepatic steatosis and modulates inflammatory and lipid metabolism–related markers under early diet-induced metabolic stress.

### L. acidophilus improves glucose tolerance and hepatic metabolic gene expression in ob/ob mice

**Figure 4.**
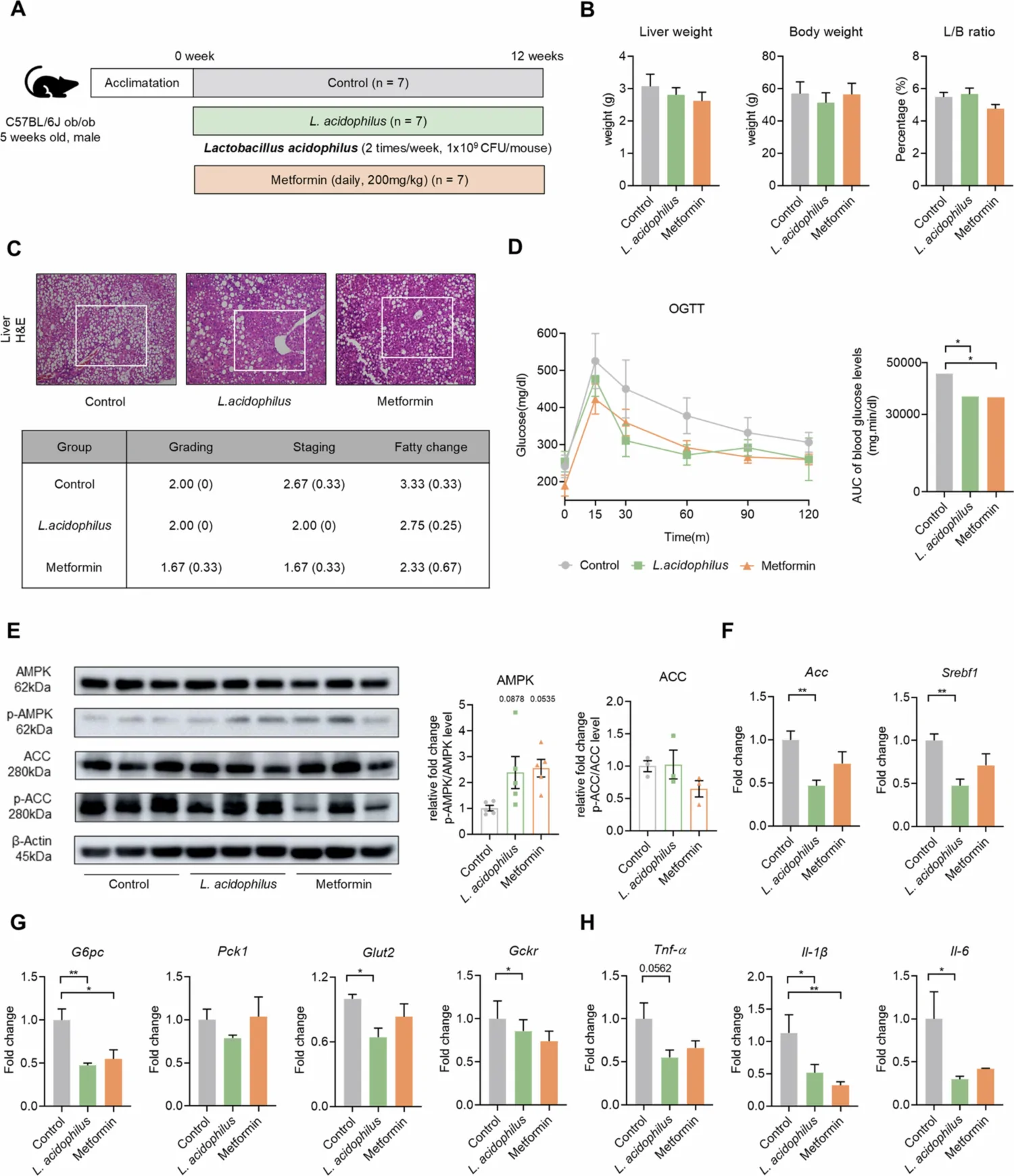
Effects of *L. acidophilus* and metformin on glucose metabolism and hepatic gene expression in ob/ob mice. (A) Experimental scheme for the ob/ob mouse model treated with *L. acidophilus* or metformin (Met). (B) Liver weight, body weight, and liver-to-body weight ratio. (C) Representative H&E-stained liver sections and corresponding grading, staging, and fatty change scores. (D) Oral glucose tolerance test (OGTT) and area under the curve (AUC) for blood glucose levels. (E) AMPK and ACCphosphorylation assessed by western blot. (F) Hepatic mRNA expression lipid metabolism–related genes, (G) gluconeogenesis and glucose transport–related genes and (H) proinflammatory genes. Values are shown as mean ± SEM. Significant differences between groups are denoted as follows *P < 0.05, **P < 0.01. Met, Metformin; AMPK, AMP-activated protein kinase; AdipoR1, Adiponectin receptor1; AdipoR2, Adiponectin receptor2 Acc, Acetyl-CoA carboxylase; *Srebp1c*, Serpine1 mRNA-binding protein 1; *G6pc*, Glucose-6-phosphatase, catalytic subunit; *Pck1*, phosphoenolpyruvate carboxykinase 1; *Glut2*, glucose transporter type 2; *Gckr*; glucokinase regulator; *Tnf-α*, Tumor necrosis factor-alpha; *Il-1β*, Interleukin 1 beta; *Il-6*, interleukin-6.

Recent studies have reported that a high proportion of patients with T2D also have MASLD.^26^ T2D and MASLD not only commonly coexist but also exhibit a synergistic and bidirectional relationship.^27^ ^,^ ^28^ Based on the metabolic effects observed in the WD-induced MASLD model, we next evaluated its metabolic effects in a genetic model of obesity and T2D. Leptin-deficient ob/ob mice are widely used to model obesity- and T2D-like metabolic dysfunction.^29^ Ob/ob mice were assigned to three groups: disease control, *L. acidophilus*-treated, and metformin-treated groups, with *n* = 7 mice in each group. The Met group was included as a positive control. Ob/ob mice received *L. acidophilus* or metformin by oral gavage for 12 weeks (Figure 4A). No marked differences in liver weight, body weight, or the liver-to-body weight ratio were observed among the groups; however, liver and body weights showed a modest downward tendency in the L. acidophilus-treated group (Figure 4B). The degree of hepatic steatosis and fibrosis tended to decrease in the *L. acidophilus* group compared with the control group, although the differences did not reach statistical significance (Figure 4C). The oral glucose tolerance test (OGTT) was performed to assess systemic glucose tolerance following glucose administration.^30^ The disease control group showed higher glucose excursion during the OGTT, whereas the AUC was significantly lower in both the L. acidophilus and metformin groups than in the control group (control, 45,732; *L. acidophilus*, 36,888; Metformin, 36,510; *P < 0.05 vs control) (Figure 4D). Serum insulin (control, 1.86 ± 1.57 ng/mL; *L. acidophilus*, 0.38 ± 0.36 ng/mL) and C-peptide (control, 1.398 ± 0.7 ng/mL; *L. acidophilus,* 0.98 ± 0.28ng/mL) levels were also decreased, but these differences did not reach statistical significance (Supplementary Figure 3A).

To further explore pathways associated with the metabolic effects of *L. acidophilus* in ob/ob mice, we assessed hepatic AMPK and ACC phosphorylation and related metabolic gene expression. AMPK is a key energy sensor that regulates glucose and lipid metabolism,^31^ ACC is one of its downstream metabolic targets involved in lipid synthesis. Western blot analysis showed a tendency toward increased AMPK phosphorylation in the *L. acidophilus*-treated group, with a similar directional pattern observed in the metformin group. In contrast, ACC phosphorylation did not show a marked increase, and inter-individual variability was observed (Figure 4E, Supplementary Figure 3B). We next examined hepatic expression of genes related to adiponectin signaling, lipid metabolism, gluconeogenesis, and inflammation. In parallel with the changes in AMPK phosphorylation, the expression of the lipogenic genes *Acc* and *Srebf1* was significantly reduced in the *L. acidophilus*-treated group, while metformin showed a nonsignificant decreasing tendency for these genes (Figure 4F). Genes related to gluconeogenesis (*G6pc*, *Pck1*, *Glut2*, *Gckr*) tended to decrease in the *L. acidophilus* group (Figure 4G). In addition, pro-inflammatory cytokine-related genes (*Tnf-α, Il-1β, Il-6*) were reduced following *L. acidophilus* treatment (Figure 4H). Together, these findings suggest that L. acidophilus treatment was associated with improved glucose tolerance and a tendency toward increased hepatic AMPK phosphorylation in ob/ob mice, accompanied by changes in genes related to hepatic lipogenesis, gluconeogenesis, and inflammation.

### L. acidophilus treatment is associated with hepatic transcriptomic modulation of lipid and glucose metabolism

**Figure 5.**
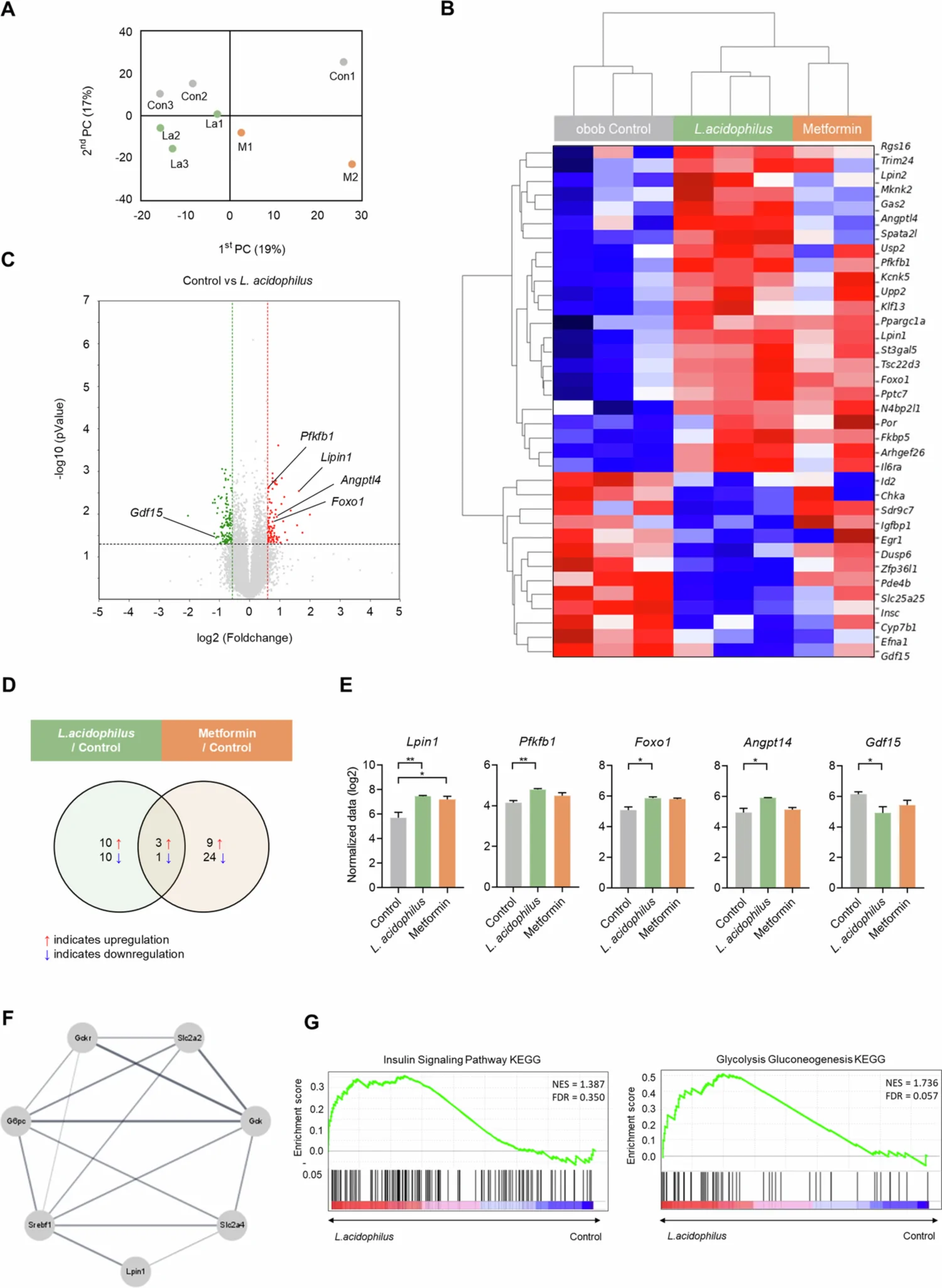
*L. acidophilus* modulates hepatic transcriptional networks linked to lipid and glucose metabolism in ob/ob mice. (A) Principal component analysis (PCA) of hepatic transcriptomes. (B) Heatmap and hierarchical clustering of differentially expressed genes (row z-score). (C) Volcano plot of hepatic differentially expressed genes between control and *L. acidophilus*-treated ob/ob mice. (D) Venn diagram showing overlap of differentially expressed genes (DEGs) in *L. acidophilus* vs control and metformin vs control comparisons. (E) Normalized RNA-seq expression levels (log₂) of representative genes (F) Gene interaction network generated using the STRING database to visualize functional associations among metabolism-related genes, highlighting nodes linked to glucose and lipid metabolic regulation (G) Gene set enrichment analysis (GSEA) of of KEGG pathways comparing *L. acidophilus*-treated mice with controls, including insulin signaling and glycolysis/gluconeogenesis pathways. Values are shown as mean ± SEM. Significant differences between groups are denoted as follows *P < 0.05, **P < 0.01. *Dusp10*, dual specificity phosphatase 10; *Gdf15*, growth differentiation factor 15; *Il6ra*, interleukin 6 receptor subunit alpha; *Fkbp5*, FKBP prolyl isomerase 5; NES, normalized enrichment score; FDR, false discovery rate.

Gene expression profiling of liver tissue was performed using QuantSeq 3′ mRNA sequencing to investigate transcriptional changes associated with *L. acidophilus* treatment. Principal component analysis (PCA) revealed distinct clustering of gene expression profiles, showing clear separation between treatment groups (Figure 5A). Consistently, hierarchical clustering of differentially expressed genes indicated that the *L. acidophilus* and metformin groups clustered more closely with each other than with the ob/ob control group (Figure 5B). Volcano plot analysis showed that a subset of genes was upregulated or downregulated in the *L. acidophilus*-treated group compared with the control group, based on the predefined fold-change and *P*-value thresholds. These findings indicate that L. acidophilus treatment was associated with hepatic transcriptomic modulation in ob/ob mice (Figure 5C).Gene Ontology (GO) enrichment analysis comparing the *L. acidophilus* and control groups identified biological processes related to lipid metabolism and signaling regulation, including positive regulation of fat cell differentiation, response to nutrient levels, and serine/threonine kinase signaling pathways (Supplementary Figure 4A). Exploratory KEGG pathway analysis further suggested the involvement of metabolism-related pathways, including insulin signaling, glucagon signaling, glycerophospholipid metabolism, and AMPK signaling, although these pathways did not reach strong significance after multiple-testing correction (Supplementary Figure 4B). These results suggest that *L. acidophilus* is associated with transcriptional programs linked to lipid metabolism, nutrient sensing, and intracellular signaling. To compare the transcriptomic effects of *L. acidophilus* and metformin, a Venn diagram analysis was conducted (Figure 5D). To further illustrate these transcriptional changes, normalized RNA-seq expression levels (log₂) of five representative metabolic genes (*Lpin1, Pfkfb1, Foxo1, Angptl4 and Gdf15)*) were compared among treatment groups (Figure 5E). Among them, *Lpin1* showed correlations with genes involved in lipogenesis and gluconeogenesis. To visualize potential functional relationships among genes implicated in these pathways, a gene interaction network was generated using the STRING database. The resulting network highlighted several core metabolic regulators, including *Srebf1*, *G6pc*, *Gck*, *Gckr*, and *Lpin1*, which were functionally connected to multiple nodes associated with glucose and lipid metabolism. Although not all these genes exhibited significant transcriptional changes, their inclusion provides contextual information rather than direct mechanistic evidence of *L. acidophilus* may co-ordinately regulate hepatic metabolic pathways (Figure 5F). Finally, gene set enrichment analysis (GSEA) was performed to identify significantly enriched pathways. The glycolysis/gluconeogenesis pathway was positively enriched in the *L. acidophilus* treated group compared with the control (NES = 1.736, FDR = 0.057), indicating a borderline trend Insulin signaling also showed positive enrichment (NES = 1.387, FDR = 0.350), although this did not reach statistical significance (Figure 5G).

### L. acidophilus modulates gut microbial composition in ob/ob mice with shared signatures across metabolic disease cohorts

**Figure 6.**
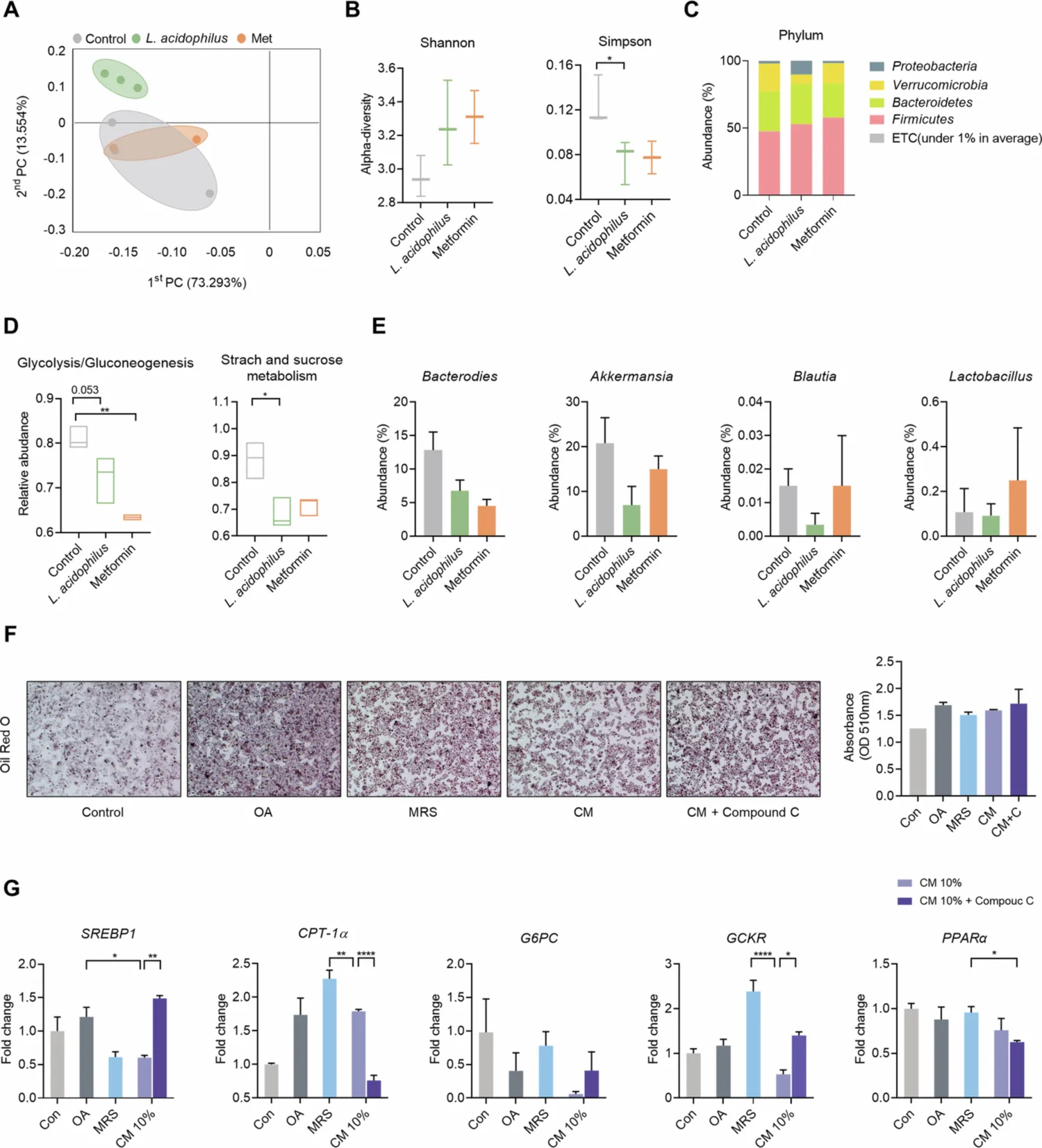
(A–E) Fecal microbiome profiling was performed by 16S rRNA gene sequencing. (A) PCoA based on *β*-diversity showing separation of microbial community structures among groups. (B) *α*-diversity indices (Shannon and Simpson) across groups. (C) Relative abundance of major bacterial phyla. (D) Predicted functional profiling based on 16S data. (E) Genus-level relative abundance of representative taxa. (F, G) HepG2 cells were challenged with OA and treated with MRS medium control, *L. acidophilus*–conditioned medium (CM, 10%), or CM co-treated with Compound. (F) Representative Oil Red O–stained images. (G) Quantification of Oil Red O staining by absorbance at 510 nm (OD510). (H) Gene expression analysis of lipid and glucose metabolism–related genes. Values are shown as mean ± SEM. Significant differences between groups are denoted as follows *P < 0.05, **P < 0.01, ***P < 0.001. PCoA, Principal coordinates analysis; OA, Oleic acid; CM, Conditioned medium.

Mice fecal samples were analyzed using 16S-based microbial taxonomic profiling to identify microbiota differences among groups. Principal coordinates analysis (PCoA) of *β*-diversity showed that the *L. acidophilus*-treated group occupied a position distinct from the ob/ob control group, indicating an overall shift in community composition (Figure 6A). Consistent with the distinct clustering observed in *β*-diversity analysis, *L. acidophilus* treatment induced noticeable changes in overall gut microbial diversity. The Shannon index exhibited a slight increasing trend in the *L. acidophilus*–treated group compared with control mice, whereas the Simpson index was significantly decreased, indicating enhanced community evenness and diversity (Figure 6B). At the phylum level, ob/ob control mice showed relatively lower *Firmicutes*, while *Bacteroidetes* abundance remained largely similar across groups (Figure 6C). Functional inference based on 16S profiles suggested that *L. acidophilus* treatment was associated with altered predicted microbial functional potential. Pathways related to glycolysis/gluconeogenesis and starch and sucrose metabolism were higher in the control group and lower after *L. acidophilus* supplementation, although these predictions reflect inferred fuctional potential rather than direct metabolic activity (Figure 6D). At the genus level, *Akkermansia*, *Bacteroides*, and *Blautia* tended to be higher in the control group and lower in the *L. acidophilus*-treated group, although these differences did not reach statistical significance. *Lactobacillus* abundance increased in the metformin group, whereas no clear change was observed between ob/ob controls and the *L. acidophilus*-treated group (Figure 6E). LEfSe analysis detected only minor differences among low-abundance taxa and did not identify dominant discriminatory signatures (Supplementary Figure 5). Overall, these results indicate that *L. acidophilus* supplementation is accompanied by modest compositional shifts and changes in diversity-related indices in ob/ob mice. The direction of these microbial changes is broadly consistent with disease-associated patterns observed in metabolic disease cohorts, supporting an association between probiotic treatment and gut microbial dysbiosis across human and experimental settings.

### L. acidophilus-conditioned media modulates hepatocyte lipid and glucose metabolic genes in association with AMPK-related signaling

To probe whether the effects of *L. acidophilus*–conditioned medium (CM) are sensitive to AMPK inhibition, HepG2 cells treated with 10% CM were co-incubated with Compound C, a pharmacological AMPK inhibitor. Oil Red O staining showed oleic acid (OA)-induced intracellular lipid accumulation, and quantification by absorbance at 510 nm revealed a slight increase in lipid staining in the CM + Compound C group relative to CM alone (Figure 6F). We next assessed expression of genes related to lipid and glucose metabolism. Compared with CM alone, co-treatment with Compound C attenuated CM-associated regulation of metabolic genes. In particular, *SREBP1* expression was significantly higher in the CM + Compound C group than in the CM group, indicating a partial reversal of CM-mediated suppression. Likewise, *CPT1α* expression was markedly reduced by Compound C in CM-treated cells compared with CM alone. Co-treatment with Compound C also altered CM-associated expression patterns of *GCKR* and *PPARα*, whereas *G6PC* showed no clear difference between CM and CM + Compound C under these conditions (Figure 6G). Similar CM- and Compound C–associated expression patterns were also observed under basal (no OA) conditions (Supplementary Figure 6). Overall, these data indicate that CM-associated gene expression responses in HepG2 cells are sensitive to pharmacological AMPK inhibition.

## Discussion

Gut dysbiosis has been widely associated with metabolic disorders, including MASLD and T2D.^32^ ^,^ ^33^ In this study, we observed that the gut microbial ecosystem exhibited compositional and functional alterations accompanying the progression of hepatic metabolic disorders. These findings support the concept that gut microbial imbalance may reflect, and potentially contribute to, metabolic deterioration during MASLD progression.^34^ To explore this further, we first analyzed fecal microbiome profiles from a human cohort stratified by liver disease severity and the presence of diabetes mellitus (DM; clinically diagnosed type 2 diabetes in this study) to identify disease- and diabetes-associated microbial signatures relevant to metabolic dysfunction. Using stepwise comparisons across MASLD/MASH progression and four-group stratification (HC, HC + DM, MASH, MASH + DM), we observed progressive diversity loss and compositional shifts in the combined disease context, and extended genus-level observations with shotgun metagenomics and multivariable association testing to prioritize *L. acidophilus* as a human microbiome-informed candidate for subsequent in vivo validation.

*L. acidophilus* supplementation partially improved intestinal barrier–related markers and lowered circulating endotoxin levels in WD–fed mice. Tight junction genes such as *Occludin* and *Claudin* were increased following *L. acidophilus* treatment, which may suggest enhanced epithelial barrier function under dysbiosis conditions.^32^ Paralleling these changes, 16S rRNA analysis revealed that *L. acidophilus* modulated the overall gut microbial structure disrupted by WD feeding. Although the causal contribution of microbiome restructuring to hepatic outcomes is not fully defined, these findings raise the possibility that *L. acidophilus* may contribute to hepatic improvement in part by improving barrier integrity and dampening microbially derived inflammatory cues.

To evaluate the potential of *L. acidophilus* across different stages of MASLD progression, we applied two additional dietary models with distinct pathophysiological characteristics. The FPC diet model recapitulates advanced steatohepatitis with fibrotic remodeling, whereas the HFD model represents an earlier stage characterized by hepatic steatosis and mild inflammation. In the HFD model, *L. acidophilus* supplementation was associated with attenuation of hepatic lipid accumulation and inflammatory changes, consistent with an effect in an earlier steatotic disease context. In contrast, in the FPC model, overt histological and serum biochemical recovery was limited, whereas changes in fibrosis-, inflammation-, and lipid metabolism-related gene expression suggested molecular-level modulation under more advanced steatohepatitis-like injury. Together with the microbial patterns observed in our clinical cohort, these finding support a possible association between *L. acidophilus* and metabolic disease-associated microbial alterations, rather than indicating uniform *Lactobacillus* enrichment across models. Furthermore, these findings suggest that the metabolic benefits of *L. acidophilus* extend beyond hepatic improvement to include the regulation of systemic glucose metabolism and insulin responsiveness, which are further explored in our diabetic model.

However, these findings should be interpreted within the context of the model-specific experimental designs. The administration frequency of *L. acidophilus* differed among animal models because each experiment was designed for a distinct disease context and intervention purpose, including therapeutic intervention in WD- and FPC diet-induced models and preventive or early-intervention settings in HFD and ob/ob models. Therefore, the magnitude of probiotic efficacy should not be directly compared across models, and the effects of *L. acidophilus* should be interpreted within each experimental context rather than as a head-to-head comparison under identical baseline conditions.

Several studies have reported hepatic metabolic effects of *L. acidophilus* in experimental liver injury and diet-induced metabolic dysfunction.^35^ Prior studies have reported that *L. acidophilus* alleviates D-galactosamine–induced hepatic injury and reduces cholesterol accumulation and steatosis in HFD models, thereby mitigating features of liver disease progression.^36^ ^,^ ^37^ Consistent with these reports, our study found that *L. acidophilus* supplementation reduced hepatic cholesterol content and attenuated lipid accumulation. Because hepatic lipid dysregulation is closely linked to insulin resistance and impaired glucose handling, these improvements likely contribute to the metabolic benefits observed in our diabetic model, where *L. acidophilus* treatment enhanced glucose tolerance and improved insulin sensitivity.

Because dysregulated hepatic lipid metabolism promotes insulin resistance and impaired glucose homeostasis, we examined *L. acidophilus* in a diabetic model.^38^ *L. acidophilus* administration reduced hepatic expression of pro-inflammatory cytokines (*Tnf-α, Il-1β, Il-6*), suggesting attenuation of inflammation-driven metabolic stress. *L. acidophilus* treatment was associated with an increasing tendency in AMPK phosphorylation, a central regulator of glucose and lipid metabolism.^39^ Because AMPK activation is known to suppress lipogenesis and enhance fatty-acid oxidation, the observed gene expression changes may be compatible with AMPK-associated metabolic regulation.^40^ *L. acidophilus*-treated mice exhibited decreased expression of lipogenic genes (*Srebf1* and *Acc*) and increased expression of the fatty acid oxidation regulator *Ppara*, with lower expression of glucose metabolism–related genes including *Pepck* and *G6pc*. Although these patterns align with AMPK-associated regulation, further mechanistic studies will be necessary to define the extent to which AMPK mediates these transcriptional responses. In hepatocyte experiments, co-treatment with Compound C attenuated CM-associated gene expression changes, indicating that at least part of the in vitro response is sensitive to pharmacological AMPK inhibition, while not establishing direct causality. Genes associated with glycogen degradation were downregulated following *L. acidophilus* treatment pointing to a shift in hepatic glucose flux.^41^ ^,^ ^42^ Collectively, these alterations indicate that *L. acidophilus* influences multiple regulatory nodes involved in hepatic lipid and glucose homeostasis.

Transcriptomic profiling further supported broad metabolic effects of *L. acidophilus*. PCA and hierarchical clustering showed partial separation of *L. acidophilus*-treated mice from diabetic controls, and differentially expressed genes included regulators involved in inflammation, lipid metabolism, and nutrient sensing. The modest enrichment of adipocyte-associated and MAPK-related gene sets suggests that *L. acidophilus* may influence transcriptional programs beyond classical AMPK-related pathways. Among the differentially expressed genes, *Lpin1* was increased. Hepatic *LPIN1* has been implicated in metabolic regulation and is frequently reduced in obesity and T2D.^43^ *LPIN1* has also been reported to associate with glucose metabolism-related gene programs,^44^ and given its established roles in lipid and glucose metabolism,^45^ the observed upregulation of *Lpin1* could represent one component of broader metabolic adaptation. Additional genes, including *Dusp10*, *Gdf15*, *Il6ra*, and *Fkbp5*, are consistent with changes in hepatic stress responses and nutrient sensing gene network analysis identified clusters of genes interconnected within hepatic glucose–lipid pathways, suggesting coordinated metabolic adaptation rather than isolated gene-specific effects. Although these genes are not established direct AMPK targets, their expression patterns may be compatible with AMPK-related metabolic adjustment without implying direct causality. Consistently, GSEA revealed a positive enrichment trend in the Glycolysis/Gluconeogenesis pathway (NES = 1.736, FDR = 0.057) and a non-significant trend in insulin signaling, indicating subtle shifts in transcriptional activity related to glucose metabolism. Given the modest statistical significance, these findings should be interpreted as supportive rather than definitive and considered alongside physiological outcomes in the diabetic model.

Consistent with the human cohort signatures described above, microbiome profiling in our mouse models similarly revealed reduced diversity and compositional disturbances under metabolic disease conditions.^46^ ^,^ ^47^ However, *Lactobacillus* abundance did not consistently increase after L. acidophilus supplementation across all models. This may reflect the fact that oral administration of a single L. acidophilus strain does not necessarily lead to a detectable increase in genus-level *Lactobacillus* abundance by 16S rRNA sequencing, particularly under distinct dietary or genetic disease contexts. Therefore, the microbiome findings should be interpreted as model-dependent microbial community modulation rather than consistent *Lactobacillus* enrichment. In addition, 16S rRNA sequencing has limited resolution below the genus level, which may preclude accurate detection of species- or strain-level engraftment of the administered probiotic.^48-50^ In the expanded human cohort, *Akkermansia* showed a nonsignificant tendency toward higher relative abundance in the HC and HC + DM groups, whereas its relative abundance decreased in *L. acidophilus*-treated ob/ob mice. These divergent findings suggest that changes in *Akkermansia* abundance may be context-dependent and could reflect compensatory, host- and microbial community-specific responses rather than a uniform protective or pathogenic role.^51^ Notably, metabolic and hepatic improvements occurred despite the lower relative abundance of *Akkermansia*, suggesting that its enrichment was not a necessary feature of the response in this model. However, because our 16S rRNA sequencing analysis assessed relative rather than absolute abundance, the biological significance of this change remains uncertain. Therefore, these human and animal findings should be interpreted cautiously rather than as direct cross-species validation.

Several limitations should be considered. First, the human microbiome analysis was cross-sectional and therefore cannot establish a causal relationship between altered *L. acidophilus* abundance and MASLD- or diabetes-associated metabolic dysfunction. Although multivariable analysis was performed to adjust for major clinical covariates, residual confounding by medication use, diet, disease duration, and other host factors cannot be excluded. Second, the animal experiments did not use a single unified model that simultaneously recapitulates both MASH and diabetes. Instead, the diet-induced WD, HFD, and FPC models were used to evaluate MASH-related hepatic phenotypes, whereas the ob/ob model was used to examine obesity- and diabetes-associated metabolic dysfunction. Therefore, the effects of *L. acidophilus* should be interpreted within each model-specific disease context, rather than as direct evidence from a single combined animal model of MASH with diabetes. Nevertheless, the use of complementary models allowed us to examine the potential effects of *L. acidophilus* across distinct components of the metabolic disease spectrum observed in the human cohort. Third, the mechanistic interpretation of AMPK signaling remains limited. Although *L. acidophilus* treatment was associated with an increasing tendency in hepatic AMPK phosphorylation and reduced ACC1 mRNA expression, these findings do not prove AMPK-dependent causality. Additional analysis of ACC phosphorylation, a canonical downstream target of AMPK, showed inter-sample variability and did not provide statistically robust evidence of AMPK-ACC pathway activation. Thus, the present findings should be interpreted as evidence of AMPK-related metabolic modulation rather than definitive mechanistic proof. Further studies using AMPK knockdown or knockout approaches are needed to clarify whether AMPK directly mediates the metabolic effects of *L. acidophilus*.

Overall, our findings highlight multifaceted metabolic actions of *L. acidophilus*, including modulation of gut microbiome composition, attenuation of hepatic inflammation, and AMPK-associated regulation of lipid and glucose pathways. These collective results support further investigation of L. acidophilus as a microbiome-informed candidate for modulating selected hepatic metabolic and microbial pathways in MASLD- and diabetes-associated metabolic dysfunction.

## Materials and methods

### Study population

A prospective cohort study was carried out between April 2017 and March 2020. The healthy control participants were recruited from a health promotion center. Eligible patients were adults aged ≥20 y with a BMI ≥23 kg/m^2^. Blood chemistry tests, including AST, ALT, creatinine, total cholesterol, *γ*-GTP, triglycerides, and HDL cholesterol, were performed in all subjects. MASLD and MASH were diagnosed based on the definitions described in the consensus statement on the new fatty liver disease nomenclature and the 2025 KASL clinical practice guidelines for metabolic dysfunction-associated steatotic liver disease.^52^ ^,^ ^53^ MASLD was defined as hepatic steatosis in the presence of metabolic dysfunction, after excluding significant alcohol consumption and other causes of chronic liver disease. The MASH group included patients with metabolic dysfunction-associated hepatic steatosis who had elevated liver enzyme levels (AST or ALT ≥ 50 IU/L) and/or histological evidence of steatohepatitis, when available. Diabetes status was determined based on medical records, including a previous clinical diagnosis of diabetes mellitus, current use of antidiabetic medication, fasting plasma glucose ≥126 mg/dL, or HbA1c ≥6.5%. Medication history, including antidiabetic agents, antihypertensive drugs, and lipid-lowering agents such as statins, was collected from medical records and was not considered an exclusion criterion. Exclusion criteria included antibiotic use within 2 months before enrollment, as well as the use of probiotics, prebiotics, or health functional foods/dietary supplements that could directly affect gut microbiota composition or liver function within 2 months before enrollment. Participants with significant alcohol consumption, viral hepatitis, autoimmune liver disease, drug-induced liver injury, malignancy, or other chronic liver diseases were also excluded. Female participants who were pregnant or lactating were also excluded. Dietary background and fibrosis-related information were reviewed based on available clinical records. Histological features in liver biopsy specimens, including hepatic steatosis, lobular inflammation, hepatocellular ballooning, and fibrosis stage, were reviewed when liver biopsy data were available. Enrolled patients and controls underwent fecal sampling and clinical analysis.

This study was controlled in accordance with ethical guidelines from the 1975 Helsinki Declaration as reflected by prior approval by the Chuncheon Sacred Heart Hospital Institutional Review Board for human research (approval no. 2016-11-134). Written informed consent was obtained from all participants prior to their participation in the study.Basic information has been registered on ClinicalTrials.gov in the public trial registry (ClinicalTrials.gov ID: NCT04339725).

### Fecal sample 16s rRNA and metagenomic analysis

To ensure consistency in fecal sample handling and microbiome data generation during the three-year study period, all fecal samples were collected, transported, and stored according to a standardized protocol. After collection, fecal samples were transported on ice and stored at −80 °C until microbial DNA extraction. Microbial DNA was extracted using the same DNA extraction kit and protocol across all samples. Library preparation, sequencing quality control, and downstream bioinformatic analyzes were conducted using a uniform workflow to minimize technical variation and batch-related effects. Genomic DNA for metagenomic analysis was isolated from 180 mg of each fecal sample using the QIAamp Fast DNA Stool Kit (QIAGEN, Hilden, Germany; Cat. No.51604) according to the manufacturer’s protocol. Sequencing libraries were constructed with the NEBNext Ultra II FS DNA Library Prep Kit for Illumina (New England Biolabs) according to the provided instructions. Library quantification was measured using the Qubit dsDNA HS Assay Kit (Thermo Fisher Scientific) and further validated by quantitative PCR using KAPA SYBR FAST qPCR Master Mix (Kapa Biosystems). Library integrity and fragment size distribution were evaluated on an Agilent 2100 Bioanalyzer with a DNA 12000 chip (Agilent Technologies). Prepared libraries were sequenced on an Illumina NovaSeq 6000 system to generate 150-bp paired-end reads.^54^

For 16S rRNA gene analysis, the V3–V4 hypervariable region was amplified using barcoded universal primers. PCR cycling was performed under the following conditions: initial denaturation at 95 °C for 5 min; 20 cycles of denaturation at 95 °C for 30 s, annealing at 55 °C for 30 s, and extension at 72 °C for 30 s; with a final extension at 72 °C for 10 min. Amplicons were purified using AMPure XP beads (Beckman Colter) and quantified using PicoGreen and qPCR prior to pooling at equimolar concentrations. The pooled library was sequenced on an Illumina MiSeq platform in accordance with the manufacturer’s guidelines. Taxonomic assignment and microbiome profiling were conducted using the 16S Microbial Taxonomic Profiling pipeline implemented in the EzBioCloud Apps platform (CJ Bioscience, Republic of Korea).

Shotgun metagenomic sequencing data were processed using the Biobakery meta-omics workflow. Quality control was performed using KneadData (v0.12.0), which invoked Trimmomatic (v0.39) for adapter removal and quality filtering. Taxonomic profiling was subsequently performed using MetaPhlAn4 (v4.0.6) with the mpa_vJun23_CHOCOPhlAnSGB_202403 database.

### Animal experiments

Six‐week‐old specific pathogen‐free C57BL/6J male mice and five‐week‐old C57BL/6J ob/ob male mice were obtained from Doo‐Yeol Biotech (Republic of Korea). Mice were housed under controlled conditions (temperature 22 ± 2 °C, 12 h light/dark cycle) with ad libitum access to food and water. Following a 1-week acclimatization period, mice were randomly allocated to the experimental groups. The order of treatment administration and outcome measurements was randomized across experimental groups to minimize potential confounding. For all in vivo experiments, the individual mouse was considered the experimental unit. Sample sizes were determined based on previous studies using comparable animal models and considerations to minimize the number of animals used.

All procedures adhered to the National Institutes of Health Guidelines for the Care and Use of Laboratory Animals and were approved by the Institutional Animal Care and Use Committee (IACUC) of Hallym University (approval no. Hallym 2021-16, 2021-63). All animal experiments were conducted and reported in accordance with the ARRIVE guidelines.

### Strain preparation

*L. acidophilus* was initially cultured on de Man, Rogosa and Sharpe (BD Difco, USA; Cat. No. 69966) at 37 °C for 24 h. A single colony was then inoculated into an optimized fermentation medium in a bench-top fermenter (MARADO-05D-PS, Bio Control & Science). Fermentation was performed at 37 °C for 18–20 h under continuous agitation (120 rpm) while maintaining the pH at 5.5–6.0 by automatic addition of 25% NaOH.

Following fermentation, bacterial cells were harvested by centrifugation at 6000 rpm for 10 min (Supra R12, Hanil). The cell pellet was concentrated 40-fold and subsequently lyophilized (Lab-Mast 10, Cooling & Heating System) according to the manufacturer’s instructions. Colony-forming units (CFU) per gram of lyophilized powder were quantified using the standard serial dilution method.

*Lactobacillus acidophilus* was cultured, harvested, and resuspended in 1X DPBS. Mice received *L. acidophilus* by oral gavage at a dose of 1 × 10^9^ CFU in 100 μL per mouse. The administration schedule was determined according to the purpose, duration, and severity of each animal model. Within each experiment, control animals received an equivalent volume of the corresponding vehicle according to the same administration schedule.

### Western diet model

For the Western diet (WD)-induced MASLD treatment model, mice were assigned to the normal chow diet group (NCD; *n* = 12), WD group (*n* = 12), or WD-fed group treated with *L. acidophilus* (WD + LA; *n* = 10). The Western diet contained 21.2 g of fat per 100 g (42% kcal), 48.5 g of carbohydrate per 100 g (42.7% kcal), and 17.3 g of protein per 100 g (15.2% kcal). *L. acidophilus* was administered twice per week during the final 8 weeks of the 16-week WD feeding period. At the end of each experimental period, mice were euthanized under isoflurane inhalation anesthesia (HanaPharm, Seoul, Korea).

### High-fat diet model

For the high-fat diet (HFD) prevention model, mice were assigned to the NCD group (*n* = 10), HFD group (*n* = 10), or HFD-fed group treated with *L. acidophilus* (HFD + LA; *n* = 9). The HFD contained 34.9 g of fat per 100 g, providing 60% of the total calories from fat. *L. acidophilus* was administered daily throughout the 18-week HFD feeding period. At the end of each experimental period, mice were euthanized under isoflurane inhalation anesthesia (HanaPharm, Seoul, Korea).

### Fructose–palmitate–cholesterol diet model

For the fructose–palmitate–cholesterol (FPC) diet-induced treatment model, mice were assigned to the NCD, FPC, or FPC-fed group treated with *L. acidophilus* (*n* = 8 per group). The FPC diet contained 29.0 g of fat per 100 g, providing 52% of the total calories from fat. The dietary fat was primarily derived from palmitic acid (C16:0), hydrogenated vegetable oil, and anhydrous milk fat, and the diet was supplemented with choline and methionine. The drinking water was supplemented with 18.9 g/L glucose and 23.1 g/L fructose. *L. acidophilus* was administered three times per week during the final 5 weeks of the experimental period. At the end of each experimental period, mice were euthanized under isoflurane inhalation anesthesia (HanaPharm, Seoul, Korea).

### ob/ob mouse model

For the ob/ob mouse model, mice were assigned to the untreated ob/ob control group, *L. acidophilus*-treated ob/ob group, or ob/ob plus metformin group (*n* = 7 per group). *L. acidophilus* was administered twice per week during the intervention period. At the end of each experimental period, mice were euthanized under isoflurane inhalation anesthesia (HanaPharm, Seoul, Korea).

### Histopathology

Liver tissues were fixed in 10% neutral-buffered formalin at room temperature for 24 h, embedded in paraffin, sectioned, and stained with hematoxylin and eosin (H&E). Histological features were evaluated using the NAFLD Activity Score (NAS) according to the NASH Clinical Research Network criteria. Steatosis and lobular inflammation were graded on a scale of 0–3 as follows: Steatosis: 0 (<5%), 1 (5–33%), 2 (34–66%), 3 (>66%), Lobular inflammation: 0 (none), 1 (1–2 foci per × 20 field), 2 (2–4 foci per × 20 field), 3 (>4 foci per × 20 field) and Hepatocyte ballooning was graded on a scale of 0–2 (0: none, 1: few ballooned cells, 2: many ballooned cells).

All biopsy specimens were reviewed by an experienced liver pathologist in a blinded manner.

### Blood chemistry test

Animal blood samples were collected and allowed to coagulate overnight at 4 °C. Serum was obtained by centrifugation at 2000 × g for 20 min at room temperature, and the supernatant was stored at –80 °C until analysis. Serum levels of AST, ALT, total cholesterol, triglycerides, and GGT were measured using an automated biochemical analyzer (KoneLab 20; Thermo Fisher Scientific).

### Enzyme linked immunosorbent assay (ELISA)

Serum insulin and C-peptide levels were quantified using a mouse insulin ELISA kit (TMB type; Shibayagi, Japan; Cat. No. AKRIN-011T) and a mouse C-peptide ELISA kit (U type; Shibayagi, Japan; Cat. No. AKRCP-031), respectively, following the manufacturer's instructions.

### Oral glucose tolerance test

An oral glucose tolerance test (OGTT) was performed 2 d before sacrifice. After a 16-h fast, blood glucose was determined by CareSens *N* (i-Sens, Inc. Korea) from the tail vein. Mice received glucose by oral gavage (2g/kg body weight; Sigma-Aldrich, USA; Cat. No. G8270) and glucose levels were measured at 0, 15, 30, 60, 90, 120 min.

### Real time—quantitative polymerase chain reaction (RT-qPCR)

Total RNA was extracted using 1 mL TRIzol Reagent (Thermo Fisher Scientific; Cat. No. 15596026) according to the manufacturer's instructions. RNA purity and concentration were assessed using a spectrophotometer. Complementary DNA (cDNA) was synthesized from 2 μg of total RNA in a final reaction volume of 20 μL using the High-Capacity cDNA Reverse Transcription Kit (Applied Biosystems, USA; Cat. No. 4368814). Quantitative real-time PCR was performed on a LightCycler 480 Real-Time PCR System (Roche, Germany) using 10 μL of SYBR Green Master Mix (Thermo Fisher Scientific; Cat. No. A25918). Primer sequences are provided in Supplementary Table 2. Gene expression levels were normalized to GAPDH, and relative expression was calculated using the 2^−ΔΔCt method.

### Western blotting

Liver tissues (20 mg) were homogenized in 200 μL RIPA lysis buffer (Thermo Scientific; Cat. No. 89901) containing 25 mM Tris-HCl (pH 7.6), 150 mM NaCl, 1% NP-40, 1% sodium deoxycholate, and 0.1% SDS. Homogenates were incubated on ice for 1 h and centrifuged at 14,000 rpm for 10 min at 4 °C. The resulting supernatants were collected, and protein concentrations were determined using a BCA Protein Assay Kit (Thermo Scientific; Cat. No. 232227). Samples were stored at –20 °C until use. Equal amounts of protein were mixed with 5X protein sample buffer (Elpisbio; Cat. No. EBA-1052), separated by SDS–PAGE, and transferred onto PVDF membranes. Membranes were blocked at room temperature for 1 h with 5% skim milk (for AMPK) or 5% BSA (for phospho-AMPK) in TBST (1 × TBS, 0.1% Tween-20). Membranes were incubated overnight at 4 °C with primary antibodies diluted at 1:100 or 1:1000; including: TLR4 (CD284, Santa Cruz), AMPKα (#2532, Cell Signaling Technology), Phospho-AMPKα (Thr172) (#50081, Cell Signaling Technology), Acetyl-CoA Carboxylase (#3662, Cell Signaling Technology), Phospho- Acetyl-CoA Carboxylase (Ser79) (#3661, Cell Signaling Technology). After washing, membranes were incubated with HRP-conjugated anti-rabbit IgG secondary antibody (Thermo Scientific; Cat. No. 31475) diluted 1:10,000 for 1 h at room temperature. Following three washes with TBST, chemiluminescent signals were developed using Immobilon Forte HRP substrate (Merck, Cat. No. WBLUF0100) at the volume recommended in the manufacturer’s instructions and visualized with a chemiluminescence imaging system (Amersham Imager 680, GE Healthcare). Band intensities were quantified using ImageJ software.

### RNA-seq microarray analysis

Total RNA isolated from mouse liver tissues was subjected to sequencing analysis by EBIOGEN Inc. (Seoul, Korea). Differentially expressed genes (DEGs) and gene ontology (GO) analyzes were performed using the ExDEGA software package (EBIOGEN Inc., South Korea). Gene set enrichment analysis (GSEA) was conducted to identify significantly enriched pathways between control and treatment groups. Protein–protein interaction networks were constructed using the STRING database (https://string-db.org/) and visualized with Cytoscape (https://www.cytoscape.org/).

### External shotgun metagenomic cohort analysis

To independently assess the genus-level findings from the study cohort, publicly available shotgun metagenomic data were obtained from curatedMetagenomicData, a Bioconductor R package providing uniformly processed (MetaPhlAn3) species-level relative abundance profiles. Nine studies comprising 2,716 samples were retrieved based on the inclusion of metabolic or liver disease groups. After excluding the metabolic syndrome group (*n* = 10) due to insufficient sample size, 2,706 samples were included in the analysis and classified into five groups: healthy controls (HC, *n* = 1,088), pre-diabetes (PreDM, *n* = 563), type 2 diabetes (T2D, *n* = 882), NAFLD (*n* = 41), and cirrhosis (*n* = 132).

To account for inter-study technical variation, study-level batch effects were adjusted by linear regression of log10-transformed relative abundances (pseudocount 10^−6^) on study identity, and the residuals were shifted to the global mean to preserve the original abundance scale. Group comparisons were performed using Wilcoxon rank-sum tests versus HC with Benjamini-Hochberg correction.

### Statistical analysis

Data are presented as the mean ± standard error of the mean (SEM). Statistical significance was set at *p* < 0.05. Group differences were analyzed using one-way ANOVA followed by Tukey’s post hoc test. All statistical analyzes were performed using GraphPad Prism 9 (GraphPad Software, San Diego, CA).

For the study cohort 16S rRNA analysis, alpha diversity and genus-level relative abundances were compared across groups using the Kruskal-Wallis test followed by pairwise Wilcoxon rank-sum tests with Benjamini-Hochberg (BH) correction. Beta diversity was assessed by principal coordinate analysis (PCoA) based on Bray-Curtis dissimilarity, and overall community differences were tested by PERMANOVA (adonis2, 999 permutations) using the vegan R package. For the internal shotgun metagenomic subset (*n* = 44), multivariable association analyzes were performed using MaAsLin2 to adjust for age, sex, BMI, PPI use, and probiotic supplementation on TSS-normalized and log-transformed data.

## Supplementary Material

Supplementary file.docx

## Data Availability

Raw sequencing data supporting the findings of this study have been deposited in the NCBI Sequence Read Archive (SRA) with the BioProject accession code PRJNA1418839. The processed data files, specifically the taxonomic abundance tables used for downstream analysis and visualization, are archived in the Zenodo repository (https://doi.org/10.5281/zenodo.18489463).
