# Supplementary material for: Effects of Lactobacillus acidophilus on hepatic and microbial dysregulation in MASLD and diabetes
Source: Gut Microbes. 2026 Sep 19;18(1):2734705. doi: 10.1080/19490976.2026.2734705 (PMC13596880; doi:10.1080/19490976.2026.2734705)
Supplement: Supplementary file 1 [file KGMI_A_2734705_SM7651.docx]

**Supplementary file**

**Effects of *Lactobacillus acidophilus* on Hepatic and Microbial Dysregulation in MASLD and Diabetes**

Hee Jin Park^a†^, In Gyu Park^a†^, Jeong Ha Park^a†^, Hyun Joon Park^a^, Jin Ju Jung ^a†^, Sang Hak Han ^b^, Ki Tae Suk^a*^

***^a^*** *Institute for Liver and Digestive Diseases, Hallym University, Chuncheon, Republic of Korea*

*^b^ Department of Pathology, Hallym University College of Medicine, Chuncheon 24253, Republic of Korea*

Supplementary table 1. Flowchart of participant selection and classification of the human cohort.

Supplementary table 2. Primer sequences used for quantitative real-time PCR

| Gene | Forward primer (5’-3’) | Reverse primer (5’-3’) |
| --- | --- | --- |
| *Gapdh* | AGGTCGGTGTGAACGGATTTG | TGGTAGAGTCATACTGGAACATGTAG |
| *Occludin* | ATGTCGCCGCGAGTATCCTC | TTTGCGGTCCTTCTCTGTGCGGTTTCTAT |
| *Claudin* | GCGGCGAGATACAGAGGTGAAAG | ACTTCTCCACATGGCACGTGAC |
| *Tnfa* | CCCAGAAAGCATGATCCGAGAT | CACCCCGAAGTTCAGTAGACAGA |
| *Il1β* | CCTCAATGGACAGAACCAACA | TCCCGACCATTGCTGTTTCCC |
| *Il6* | CGGAGAGGAGACTTCACAGAG | CAGGAAGGCAGTGGATCAGAGA |
| *Ifny* | TGACATGAACGCTACACACTGC | TCAGGCAGCGACTCCTTTTCC |
| *Cxc12* | ACAGAGCTCGAGTGTGAGTGAC | GTTAGGGTCTTTGCCAGGTCAG |
| *Cxc110* | GGAATCAAGACATCCCACACTTG | TCTTGGATCTGCCATGTGAAGA |
| *Cpt1a* | GAAGAGGTTCTCGGAGAGTTT | TCGGTGAGCAGAGTTCTTCGG |
| *Pparα* | AGAGATGCCAGCGAGAGGTGATT | CGCGAGTGGTCTTCTGAGTGTT |
| *Srebp1c* | TACTTCTTCTGGCCTGCACC | TCAGGTGGTGGAGGTCGAACCAA |
| *Cd36* | AGGCTGTTGTGGAAGTGGATGA | GGTTCTTCTCCAGGTCATCTGTC |
| *Pparγ* | CCAAGAATGTCGTGTGCTGATG | GTTCTTCAGCCAACCAGGTCAC |
| *Fas* | AACCGTGGTGTTGGCATCA | AGCTCGGGTGACTTCTTCGT |
| *Acc* | AACATGGTGACCCAGAGGAG | ACTTCCCGGCACTAGAAACCA |
| *Gck* | TACACACCTGCTCTGATGGC | TGGTGCCACAGGTTCATTC |
| *Acs* | GAGCCAGTTCAAGGTGCTCA | AGCGAGAACAAAGCAAGGAGG |
| *Pi3k* | GAGACAGGACGAGGAGGAGTGAG | TGGCAGTGGGCAAAGACAGT |
| *Akt* | ATGGACTTCTGCTGCTGAGATG | CAGGCTGTGATGGCGGATGAG |
| *Foxo1* | TGAGGGATGGAAGAGCGAGTG | GACGCCGATGGAGATGTTGAG |
| *Pepck* | TCCTTCAGCTGCACCATGTG | GAAACTGGAAGGTGACAGCAG |
| *G6pc* | AGCGGATGGTGACCATCTAC | TACCAGCCACGATGACCAAAC |
| *G1nus* | GTCCAGATGAAGATGTGGTGGTCT | GTCACAGGGCAGGCCCAAGATAC |

Supplementary figure 1. Species-level Lactobacillus profiling supports *L. acidophilus* selection in the human cohort. (A) Genus-level microbial composition heatmap. Values are presented as Z-scores, and hierarchical clustering was performed based on genus-level abundance patterns. (B) *Lactobacillus* Species Composition (Stacked Bar Plot). (C) Multivariable Linear Model (MaAsLin2). (D) Clinical Correlation (Spearman).

Supplementary figure 2. Genus-level changes in representative gut-associated taxa following *L. acidophilus* supplementation in WD–fed mice.

Supplementary Figure 3. Additional metabolic and AMPK-related analyses in ob/ob mice.

(A) Serum insulin and C-peptide levels in ob/ob mice. (B) AMPK phosphorylation assessed by western blot

Supplementary figure 4. Functional enrichment analysis after *L. acidophilus* treatment.

(A) Gene Ontology (GO) biological process enrichment analysis for genes differentially expressed in the *L. acidophilus* group compared with controls. (B) Exploratory KEGG pathway analysis of differentially expressed genes. Dot size represents the number of genes mapped to each pathway, and dot color indicates the adjusted P value.

Supplementary figure 5. LEfSe-identified low-abundance taxa in ob/ob mice

Supplementary figure 6. Effects of La-conditioned medium on metabolic gene expression in HepG2 cells under no OA conditions.
